# Supplementary material for: The SSVEP tracks attention, not consciousness, during perceptual filling-in
Source: eLife. 2020 Nov 10;9:e60031. doi: 10.7554/eLife.60031 (PMC7682990; doi:10.7554/eLife.60031)
Supplement: Transparent reporting form [file elife-60031-transrepform.docx]

***eLife’s* transparent reporting form**

We encourage authors to provide detailed information *within their submission* to facilitate the interpretation and replication of experiments. Authors can upload supporting documentation to indicate the use of appropriate reporting guidelines for health-related research (see [EQUATOR Network](http://www.equator-network.org/%20)), life science research (see the [BioSharing Information Resource](https://biosharing.org/)), or the [ARRIVE guidelines](http://www.plosbiology.org/article/info:doi/10.1371/journal.pbio.1000412) for reporting work involving animal research. Where applicable, authors should refer to any relevant reporting standards documents in this form.

If you have any questions, please consult our Journal Policies and/or contact us: [editorial@elifesciences.org](mailto:editorial@elifesciences.org).

**Sample-size estimation**

- You should state whether an appropriate sample size was computed when the study was being designed
- You should state the statistical method of sample size computation and any required assumptions
- If no explicit power analysis was used, you should describe how you decided what sample (replicate) size (number) to use

Please outline where this information can be found within the submission (e.g., sections or figure legends), or explain why this information doesn’t apply to your submission:

The effect size of our main effect of interest – the SSVEP generated by a dynamically texture-tagged invisible target - was previously unknown. As such, no explicit power analyses were used. Instead, we decided to double the amount of individual data collected per participant in comparison to our previous SSVEP paper, which used a similar method (Davidson et al., 2020). In addition, we chose to calibrate each individual’s experimental parameters to optimize the strength of SSVEPs prior to their experimental session. No sample size estimation information is supplied with this submission, but the details of our pre-experimental SSVEP calibration procedure are provided in the Methods, as well as Figure 1, and Figure 1- Figure Supplement 1.

Reference:

Davidson, M. J., Graafsma, I. L., Tsuchiya, N., & van Boxtel, J. (2020). A multiple-response frequency-tagging paradigm measures graded changes in consciousness during perceptual filling-in. *Neuroscience of Consciousness*, *2020*(1), niaa002.

**Replicates**

- You should report how often each experiment was performed
- You should include a definition of biological versus technical replication
- The data obtained should be provided and sufficient information should be provided to indicate the number of independent biological and/or technical replicates
- If you encountered any outliers, you should describe how these were handled
- Criteria for exclusion/inclusion of data should be clearly stated
- High-throughput sequence data should be uploaded before submission, with a private link for reviewers provided (these are available from both GEO and ArrayExpress)

Please outline where this information can be found within the submission (e.g., sections or figure legends), or explain why this information doesn’t apply to your submission:

- Each experiment was performed once per participant.

- Participant and trial exclusions were based on behavioral reports during experimental trials. Specifically, within every 1-minute trial, we physically removed at least one of the attended targets from screen. As participants were required to monitor these targets for disappearance, these removals served to estimate participant engagement with the task. When participants failed to identify these removal events, that trial was excluded from analysis. If, overall, participants failed to reliably report on these events throughout their experimental session, they were excluded entirely. The analysis performed and criteria used to determine these exclusions are detailed in our Methods, Figure 1 – Figure Supplement 1, and Figure 1 – Figure Supplement 2.

**Statistical reporting**

- Statistical analysis methods should be described and justified
- Raw data should be presented in figures whenever informative to do so (typically when N per group is less than 10)
- For each experiment, you should identify the statistical tests used, exact values of N, definitions of center, methods of multiple test correction, and dispersion and precision measures (e.g., mean, median, SD, SEM, confidence intervals; and, for the major substantive results, a measure of effect size (e.g., Pearson's r, Cohen's d)
- Report exact p-values wherever possible alongside the summary statistics and 95% confidence intervals. These should be reported for all key questions and not only when the p-value is less than 0.05.

Please outline where this information can be found within the submission (e.g., sections or figure legends), or explain why this information doesn’t apply to your submission:

Throughout this manuscript, each statistical test is identified in the main text, and justified in the methods. Statistical tests associated with figures are also named in the figure legends. We note that when correcting for multiple-comparisons, we predominantly have used non-parametric cluster-based permutation tests. For some of these tests, the observed value (our test-statistic of interest), falls outside the entire null-distribution obtained via cluster-based permutations. When this happens, we report the result as p-cluster < .001. At all other times we report the exact p-values. Other analyses include FDR corrections for multiple comparisons (e.g. Figure 2-Supplement 2, Figure 3), and in these cases we report the adjusted p-values. For our supplementary behavioral analyses (Figure 2 – supplement 1) we report the median and the 95% Confidence intervals, and for our SNR-time course results we include the SEM, corrected for within-participant comparisons.

We also report non-significant p-values that relate to our key questions, and measures of dispersion (e.g. SD) where possible. For our model comparisons, we report the effect size (likelihood ratio tests), and exact p-values.

(For large datasets, or papers with a very large number of statistical tests, you may upload a single table file with tests, Ns, etc., with reference to sections in the manuscript.)

**Group allocation**

- Indicate how samples were allocated into experimental groups (in the case of clinical studies, please specify allocation to treatment method); if randomization was used, please also state if restricted randomization was applied
- Indicate if masking was used during group allocation, data collection and/or data analysis

Please outline where this information can be found within the submission (e.g., sections or figure legends), or explain why this information doesn’t apply to your submission:

No allocation to separate experimental groups was performed, as this was a within subject design. We primarily compare EEG data when reporting on different numbers of invisible targets.

**Additional data files (“source data”)**

- We encourage you to upload relevant additional data files, such as numerical data that are represented as a graph in a figure, or as a summary table
- Where provided, these should be in the most useful format, and they can be uploaded as “Source data” files linked to a main figure or table
- Include model definition files including the full list of parameters used
- Include code used for data analysis (e.g., R, MatLab)
- Avoid stating that data files are “available upon request”

Please indicate the figures or tables for which source data files have been provided:

All the raw data, and analysis code have been uploaded to a publicly available repository on the open science framework: <https://osf.io/hs7fn/>

All source data used in the final figures has also been provided.
